# ﻿Pollen morphology of the genera *Hidalgoa* and *Dahlia* (Coreopsideae, Asteraceae): implications for taxonomy

**DOI:** 10.3897/phytokeys.199.79501

**Published:** 2022-06-13

**Authors:** Erandi Sánchez-Chávez, Andrew Vovides, Victoria Sosa

**Affiliations:** 1 Biología Evolutiva, Instituto de Ecología, A.C. Carretera Antigua a Coatepec 351, 91073 El Haya, Xalapa, Veracruz, Mexico Biología Evolutiva, Instituto de Ecología Veracruz Mexico

**Keywords:** Asteraceae, *Dahlia*, hexacolporate, *Hidalgoa*, pollen morphology, tricolporate

## Abstract

*Hidalgoa* and *Dahlia* are two closely related genera in Asteraceae, tribe Coreopsideae whose limits need to be clarified. Pollen morphology has been useful for delimitation at the genus level in this family. To better define these genera, the morphology of pollen grains was observed and measured using light and scanning electron microscopy. The pollen grains of 25 species of *Dahlia* and *Hidalgoa* were acetolyzed and analyzed. Pollen is tricorporate in most of the species studied, although in a few species in *Dahlia*, grains were found to be hexacolporate. The most outstanding differentiating characters among species of *Dahlia* and *Hidalgoa* are colpus length (greater in *Hidalgoa*) and shape of spines (conical in *Hidalgoa*). In addition, lalongate ora are larger in *Hidalgoa* than in *Dahlia*. A PCA analysis of thirteen pollen characters, identified species of *Hidalgoa* in a discrete group and *Dahliacuspidata* as an outlier. These distinctive attributes in pollen morphology support the idea that pollen morphology is useful for delimitation at the generic level in the *Dahlia* clade. Further evidence from other sources, genetic or anatomical, might contribute to demarcating *Dahlia* and *Hidalgoa*, and provide insight into the family’s evolutionary history.

## ﻿Introduction

**Figure 1.**
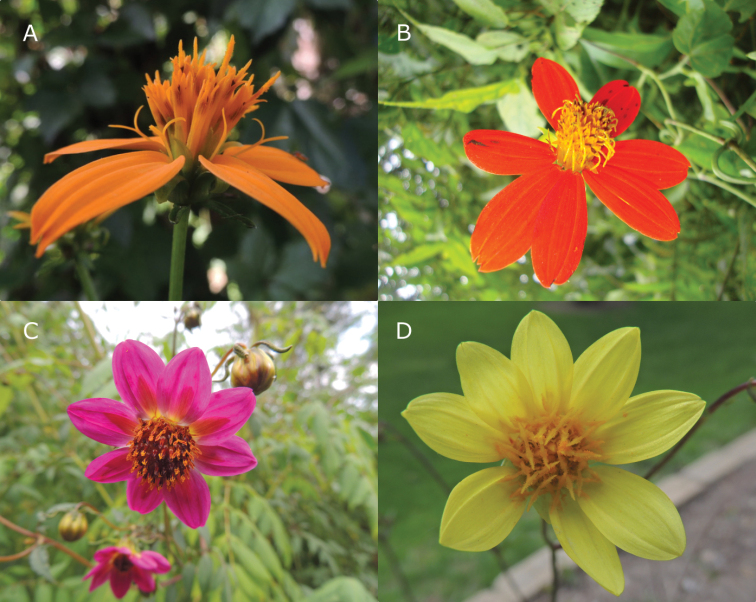
Morphological variation in *Dahlia* and *Hidalgoa* species **A***Hidalgoapentamera***B***Hidalgoauspanapa***C***Dahliamoorei***D***Dahliamixtecana*. Photos by E. Sánchez-Chávez (**A, C, D**) and Andrés Ortiz (**B**).

*Hidalgoa* La Llave and *Dahlia* Cav. are two closely related genera in tribe Coreopsideae of the Asteraceae (Sørensen 1969; Turner 2010; Sánchez-Chávez et al. 2019). *Hidalgoa* comprises four accepted species (Panero 2007; Turner 2010), with *H.ternata* La Llave having the most widespread distribution, from Mexico to northern South America. The rest have restricted distributions. *H.pentamera* Sherff and *H.uspanapa* B.L. Turner are endemic to southeastern Mexico, and *H.werklei* Hook.f. is distributed in Costa Rica and in the Andean region of Colombia. Habitats for the species in *Hidalgoa* are cloud forests, mainly in microhabitats associated with rivers and very humid places. Remarkably, plants of *Hidalgoa* are vines, climbing onto vegetation by twisting petioles. The heads of *Hidalgoa* have five to twelve pistillate, fertile ray florets, and functionally staminate disc florets (Fig. 1A, B). Cypselae are compressed, with two apical lateral cusps (Panero 2007; Crawford et al. 2009; Turner 2010; Pruski and Robinson 2015).

*Dahlia* includes 40 species, of which 37 are endemic to Mexico (Villaseñor and Redonda-Martínez 2018; Carrasco-Ortiz et al. 2019; Reyes-Santiago et al. 2019), distributed mostly in pine and oak forests (Carrasco-Ortiz et al. 2019). Based on their chromosome numbers and morphological descriptors such as life form, size, shape, and segmentation of the compound leaves, four sections were recognized in *Dahlia* (Sørensen 1969): sect. Epiphytum with a single epiphytic species (*D.macdougallii* Sherff), sect. Pseudodendron with three suffrutescent species, sect. Entemophyllon with eight suffrutescent species with solid petioles, and sect. Dahlia with 28 herbaceous species with hollow petioles (Suppl. material 1: Table S1). With the exception of the epiphytic species, the rest possess tubers, and a few are rupicolous. Some of the species in sect. Entemophyllon that live for more than one season, have stems that become quite woody and give the full-grown plants a shrubby aspect. In *Dahlia* the ray florets can be neutral, pistillate fertile or sterile, while the disk florets are hermaphroditic, and vary in number from 15 to 170 (Fig. 1C, D) (Sørensen 1969; Pruski and Robinson 2015). Cypselae are compressed, linear to spatulate, sometimes shallowly tuberculate, with pappus absent or consisting of 2(5) small teeth, weak filiform, sometimes elongated to 1 mm caducous bristles (Sørensen 1969; Panero 2007; Pruski and Robinson 2015).

*Hidalgoa* was thought to be closely related to the genera *Fitchia* Hook. f, *Moonia* Arn., *Oparanthus* Sherff, and *Petrobium* R. Br., for sharing the character of functional male disc florets (Ryding and Bremer 1992). The similar floral morphology of *Hidalgoa* and *Dahlia* was suggested to be the result of a close relationship (Sørensen 1969; Turner 2010). Furthermore, a previous phylogenetic molecular study that included species of *Dahlia* and *Hidalgoa* found that the latter was embedded in a large clade with *Dahlia*; species of the two genera formed a well-supported monophyletic group (Sánchez-Chávez et al. 2019). However, taxonomic decisions will not be made until additional anatomical and palynological characters, as well as further molecular data, can be analyzed.

In spite of the uniform pollen morphology in the genera of Asteraceae, some pollen characters have been shown to be useful for supporting recognition and delimitation in numerous genera (El-Ghazaly and Anderberg 1995). Some examples of differentiating characters in the taxa of this family are the polar diameter and dimensions of the colpus and endoaperture in *Viguiera* Kunth (Magenta et al. 2010) and in *Xanthium* L., the length and number of spines and the number of columellae (Coutinho et al. 2020). Sexine thickness, the type of aperture, and spine dimensions are the differentiating traits for species of *Stilpnopappus* and *Strophopappus* (Carrijo et al. 2013), as is pollen surface ornamentation in the complex *Phaeostigma* of the genus *Ajania* (Huang et al. 2017). Pollen type and pollen grain shape are taxonomically useful for distinguishing species and genera of the subtribe Lepidaploinae (Marques et al. 2021).

The pollen in tribe Coreopsideae is helianthoid (exine with columellae containing internal foramina and clearly caveate), predominantly spheroidal, tricolporate, echinate, with internal foramina, full cavea present, and endexine much thicker than the foot layer (Blackmore et al. 2009).

Pollen morphology has not been analyzed in detail for the species of *Hidalgoa* or *Dahlia*. Previous palynological research by Wodehouse (1929) on four *Dahlia* species identified the presence of six apertures (hexacolporate) in pollen grains. This attribute constitutes a notable exception within the family. Further studies examined and described the meiotic stages of the pollen mother cell in *Dahlia* to determine the development of these apertures (Wodehouse 1930).

The aims of this study are to compile and compare pollen morphology of the species of *Hidalgoa* and *Dahlia* to identify informative characters and understand the relationships and limits of these taxa.

## ﻿Materials and methods

Twenty-five samples of pollen grains were obtained from herbarium specimens deposited in the IBUG (Instituto de Botánica de la Universidad de Guadalajara) and XAL (Instituto de Ecología, A. C.) herbaria. Vouchers of specimens are included in Table 1.

**Table 1.** Studied species of *Hidalgoa* and *Dahlia* for analyzing pollen grains, indicating their voucher and the herbarium in which they were deposited. Herbarium acronyms are according to Index Herbariorum.

Species	Locality	Collector	Herbarium
*Dahliaatropurpurea* P.D. Sørensen	Guerrero	A. Castro C. 2251	IBUG
*Dahliaaustralis* (Sherff) P.D. Sørensen	Puebla	A. Rodriguez C. 6491	IBUG
*Dahliabarkerae* Knowles & Westc.	Jalisco	A. Castro C. 2304	IBUG
*Dahliabrevis* P.D. Sørensen	México	A. Rodriguez C. 5869	IBUG
*Dahliacampanulata* Saar, P.D. Sørensen & Hjert.	Oaxaca	A. Rodriguez C. 6495	IBUG
*Dahliacoccinea* Cav.	Jalisco	A. Rodriguez C. 7490	IBUG
*Dahliacordifolia* (Sessé & Moc.) McVaugh	Guerrero	A. Rodriguez C. 5224	IBUG
*Dahliacuspidata* Saar, P.D. Sørensen & Hjert.	Guanajuato	E. Ventura 9581	IBUG
*Dahliadissecta* S. Watson		A. Rodriguez C. 6412	IBUG
*Dahliaimperialis* Roezl ex Ortgies	Chiapas	A. Rodriguez C. 6983	IBUG
*Dahlialinearis* Sherff	Guanajuato	E. Ventura 6143	IBUG
*Dahliamerckii* Lehm.		L. Gutierrez s/n	IBUG
*Dahliamollis* P.D. Sørensen	Hidalgo	A. Rodriguez C. 6414	IBUG
*Dahlianeglecta* Saar	Hidalgo	A. Rodriguez C. 6466	IBUG
*Dahliaparvibracteata* Saar & P.D. Sørensen	Guerrero	A. Rodriguez C. 6092	IBUG
*Dahliapugana* Aarón Rodr. & Art. Castro	Jalisco	A. Rodriguez C. 7731	IBUG
*Dahliarudis* P.D. Sørensen		A. Ma. Hernández 12	XAL
*Dahliarupicola* P.D. Sørensen	Durango	A. Rodriguez C. 6133	IBUG
*Dahliascapigera* Knowles & Westc.	Queretaro	E. Gonzalez P. 560	IBUG
*Dahliasorensenii* H.V. Hansen & Hjert.		J. Suárez J. 584	IBUG
*Dahliaspectabilis* Saar & P.D. Sørensen	San Luis Potosí	A. Rodriguez C. 6352	IBUG
*Dahliatenuicaulis* P.D. Sørensen	Jalisco	M. Chazaro B. 5736	IBUG
*Dahliawixarika* Art. Castro, Carr.-Ortiz & Aarón Rodr.	Jalisco	A. Castro C. 2983	IBUG
*Hidalgoapentamera* Sherff	Veracruz	E. Sánchez-Chávez 28	XAL
*Hidalgoaternata* La Llave	Veracruz	T. B. Croat 25505	XAL

Pollen grains were acetolyzed according to the methodology of Erdtman (1960), and for difficult material in which compounds formed thin coats on the grains that interfered during the scanning process, the suggestions of Fonnegra (1989) were implemented. The grains were immersed in glacial acetic acid for 24 hours before acetolysis and then transferred to the acetolysis mixture for 1 to 6 hours and the temperature of the water bath was raised to 96 °C. For light microscopy (LM), the pollen grains were mounted in glycerol jelly, sealed, and then examined with a Carl Zeiss Fomi III Optical Microscope, equipped with a Cannon Power Shot G9 digital camera. Permanent slides were deposited in the Palynological Laboratory of the Instituto de Ecología, A. C. The following pollen measurements were obtained from 25 grains per sample: polar axis, equatorial diameter, exine thickness, colpus length, colpus width, ora width, ora length, spinae length, spine width at base and number of apertures.

To observe the pollen with a scanning electron microscope (SEM), acetolyzed pollen grains were washed in ethanol and later in water. Grains were sputter-coated with gold and observed using a Carl Zeiss EVO-50 scanning electron microscope. The terminology of Halbritter et al. (2018) was used, and for pollen structure the terminology of Erdtman (1969) was followed. Number of spines/100 μm^2^, colpus end, base of spine and pollen surface ornamentation were described for five grains per sample. Final morphological data are presented in Table 2.

**Table 2.** Pollen attributes analyzed on the studied species of *Hidalgoa* and *Dahlia*. The values given in exine, colpus, ora, spine are averages. Cl colpus length, Cw colpus width, Ow Os width, Ol Os length, Swab Width at base.

Specie	Polar axis (P)(μm)			Equatorial diameter (E)(μm)			P/E	Pollen shape	Number of apertures	Exine (μm)	Colpus (μm)			Os (μm)		Spine		Number of spines/100 μm^2^	Base of spine
	Min	Max	Mean	Min	Max	Mean					Cl	Cw	Copus ends	Osl	Ow	Length (μm)	Swab (μm)		
*Dahliaatropurpurea*	28.18	32.70	30.43	28.85	32.46	30.92	0.98	Oblate-spheroidal	Tricolporate	2.88	3.79	3.12	acute	2.28	2.55	7.80	5.99	7–8	distended
*Dahliaaustralis*	25.38	29.98	28.43	23.88	30.49	28.32	1.00	Spheroidal	Tricolporate	2.63	3.01	3.08	acute	2.56	2.05	6.01	4.51	6–8	distended
*Dahliabarkerae*	30.62	36.15	33.78	30.86	34.82	33.34	1.01	Prolate-spheroidal	Tricolporate	3.58	6.38	5.63	acute	2.20	3.09	7.55	7.21	4–5	distended
*Dahliabrevis*	26.41	32.33	29.04	25.53	32.58	29.41	0.99	Oblate-spheroidal	Tricolporate	1.81	4.83	4.74	obtuse	2.06	2.93	7.37	5.79	7–10	distended
*Dahliacampanulata*	30.61	34.72	32.84	30.50	34.48	32.96	1.00	Spheroidal	Tricolporate	2.24	4.34	1.54	obtuse	1.44	1.92	7.35	6.99	5–7	smooth
*Dahliacoccinea*	29.42	39.47	34.74	31.66	39.83	34.79	1.00	Spheroidal	Tricolporate	3.05	5.42	4.53	obtuse	3.18	2.71	9.81	8.17	4–5	distended
*Dahliacordifolia*	27.80	32.03	29.64	28.00	33.46	30.61	0.97	Oblate-spheroidal	Tricolporate	2.96	5.75	4.26	obtuse	2.32	2.74	7.87	6.64	6–7	smooth
*Dahliacuspidata*	31.21	35.48	33.16	31.63	37.05	34.44	0.96	Oblate-spheroidal	Hexacolporate/ Tricolporate	1.64	8.61	6.22	obtuse	4.75	5.24	9.07	6.94	4–6	distended
*Dahliadissecta*	28.34	32.50	30.55	27.72	32.21	30.31	1.01	Prolate-spheroidal	Hexacolporate/ Tricolporate	2.88	3.80	2.95	obtuse	2.18	2.35	7.33	6.57	5–7	smooth
*Dahliaimperialis*	25.95	30.64	28.04	25.78	31.50	28.94	0.97	Oblate-spheroidal	Hexacolporate/ Tricolporate	1.98	3.85	2.93	obtuse	2.17	2.93	7.46	5.95	6–8	smooth
*Dahlialinearis*	29.83	33.79	31.82	29.85	34.99	32.68	0.97	Oblate-spheroidal	Tricolporate	2.19	3.98	4.44	obtuse	2.42	3.70	5.29	5.59	8–10	smooth
*Dahliamerckii*	26.19	31.65	28.54	28.64	34.78	31.61	0.90	Oblate-spheroidal	Hexacolporate/ Tricolporate	3.05	4.59	3.16	obtuse	1.45	2.75	6.97	6.25	7–8	distended
*Dahliamollis*	26.59	30.26	28.38	28.14	32.36	29.93	0.95	Oblate-spheroidal	Tricolporate	1.88	4.14	2.42	obtuse	2.20	2.42	7.79	6.27	5–7	distended
*Dahlianeglecta*	32.72	37.48	35.06	30.75	37.77	35.77	0.98	Oblate-spheroidal	Tricolporate	4.20	4.81	3.34	obtuse	2.35	3.03	6.82	6.80	4–5	distended
*Dahliaparvibracteata*	29.58	32.88	31.29	28.73	32.67	31.57	0.99	Oblate-spheroidal	Hexacolporate/ Tricolporate	3.11	4.82	2.97	obtuse	2.05	2.97	8.97	6.86	6–7	distended
*Dahliapugana*	29.11	33.23	31.09	29.56	33.94	31.78	0.98	Oblate-spheroidal	Tricolporate	3.42	4.41	4.08	obtuse	2.68	2.23	6.22	6.35	6–7	distended
*Dahliarudis*	30.08	35.73	33.29	33.40	36.78	34.94	0.95	Oblate-spheroidal	Hexacolporate/ Tricolporate	3.06	4.19	2.50	acute	2.41	2.50	8.19	7.74	6–7	narrower
*Dahliarupicola*	28.20	35.37	31.16	26.92	32.40	30.08	1.04	Prolate-spheroidal	Tricolporate	1.71	3.36	2.76	obtuse	2.41	2.76	8.36	5.99	6–7	distended
*Dahliascapigera*	26.68	32.66	30.56	27.01	31.57	29.38	1.04	Prolate-spheroidal	Tricolporate	2.15	6.58	2.65	acute	2.68	2.65	7.90	6.09	4–5	narrower
*Dahliasorensenii*	28.87	34.80	31.90	30.73	36.42	33.90	0.94	Oblate-spheroidal	Hexacolporate/ Tricolporate	1.68	5.17	2.40	obtuse	1.73	2.40	9.07	6.12	4–5	narrower
*Dahliaspectabilis*	27.71	34.07	30.33	25.20	32.12	30.11	1.01	Prolate-spheroidal	Tricolporate	2.46	4.72	2.05	obtuse	1.54	2.05	8.57	6.00	6–8	distended
*Dahliatenuicaulis*	28.54	32.84	31.03	31.58	35.37	33.01	0.94	Oblate-spheroidal	Tricolporate	2.82	4.19	4.94	obtuse	2.56	2.79	9.22	6.43	7	distended
*Dahliawixarika*	27.85	33.23	30.12	27.24	32.07	30.15	1.00	Spheroidal	Tricolporate	2.42	3.36	2.63	obtuse	2.95	2.63	7.44	6.01	7–9	narrower
*Hidalgoapentamera*	25.73	30.35	27.97	25.10	30.27	26.94	1.04	Prolate-spheroidal	Tricolporate	1.78	14.97	3.29	acute	2.06	9.98	5.47	6.09	4–5	narrower
*Hidalgoaternata*	28.01	34.07	30.36	26.45	32.08	29.63	1.02	Prolate-spheroidal	Tricolporate	1.71	14.55	3.34	acute	2.89	8.21	5.80	4.63	4	narrower

A matrix based on thirteen pollen characters was constructed. To estimate the quantitative variation within *Dahlia* and *Hidalgoa*, each character was measured and the average for each species was estimated. Qualitative characters were coded with the following states: number of apertures (tricolporate:0/ hexacolporate:1), colpus end (obtuse:0/ acute:1), base of spine (narrower:0/ distended:1) lalongate ora (absent:0/ present:1; present/absent:2) (Suppl. material 1: Table S2). A principal component analysis (PCA) was run in R (R Core Team. 2019) to evaluate the contribution of each pollen variable to the affiliation of species (Table 3). Graphical representation displayed distribution of thirteen pollen characters.

**Table 3.** Palynological characters used in the multivariate analysis of *Hidalgoa* and *Dahlia* species. The contribution of every character for Axis 1 and Axis 2 is indicated (see Fig. 4).

		Character	Axis 1	Axis 2
1	Pa	Polar axis (μm)	1.63	23.53
2	Et	Exine thickness (μm)	6.59	2.30
3	Cl	Colpus length (μm)	25.18	1.19
4	Cw	Colpus width (μm)	8.88	10.75
5	Ow	Os width (μm)	22.82	1.34
6	Ol	Os length(μm)	3.75	11.48
7	Sl	Spinae length (μm)	7.98	10.97
8	Swab	Spine width at base (μm)	5.48	20.32
9	Na	Number of apertures	1.06	2.37
10	Ns	Number of spines/100 μm^2^	4.49	11.70
11	Ce	Colpus ends	8.81	0.25
12	Sb	Base of spine	3.14	1.55
13	Osl	Os lalongate	0.20	2.23

## ﻿Results

**Figure 2.**
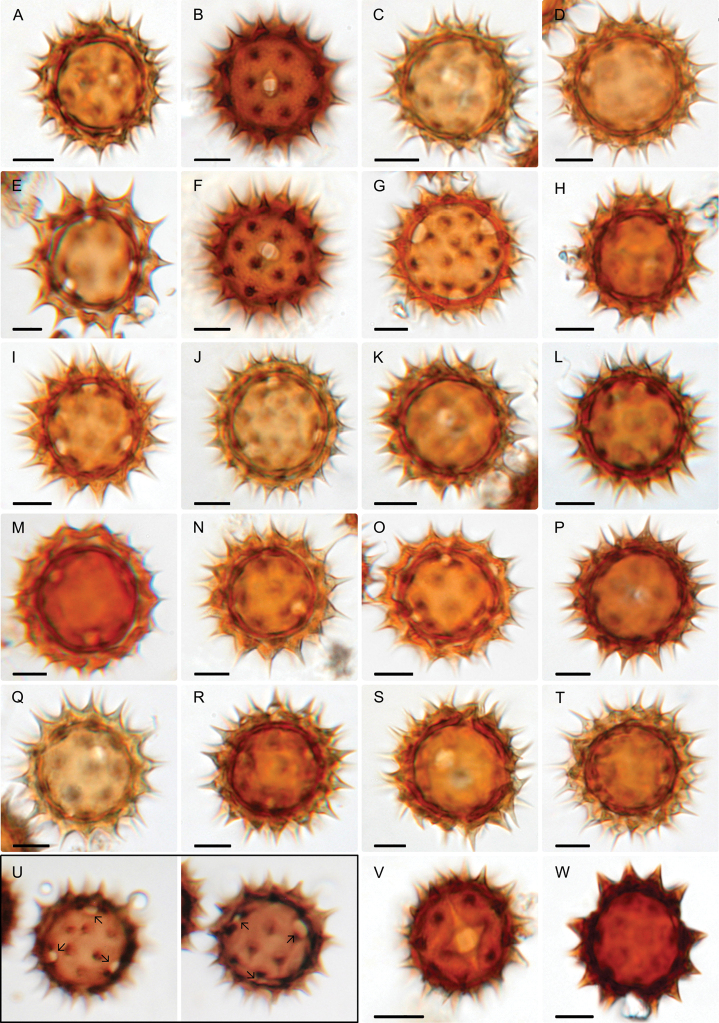
Pollen grains of *Dahlia* and *Hidalgoa* observed with light microscopy (LM) **A***D.australis***B***D.barkerae***C***D.brevis***D***campanulata***E***D.coccinea***F***D.cordifolia***G***D.cuspidata***H***D.dissecta***I***D.imperialis***J***D.linearis***K***D.merckii***L***D.mollis***M***D.neglecta***N***D.parvibracteata***O***D.pugana***P***D.rudis***Q***D.rupicola***R***D.scapigera***S***D.sorensenii***T***D.spectabilis***U***D.rudis*, pollen grain hexacolporate with three apertures on one hemisphere and three on the other hemisphere **V***H.ternata***W***H.pentamera*. Scale bars: 10 μm.

**Figure 3.**
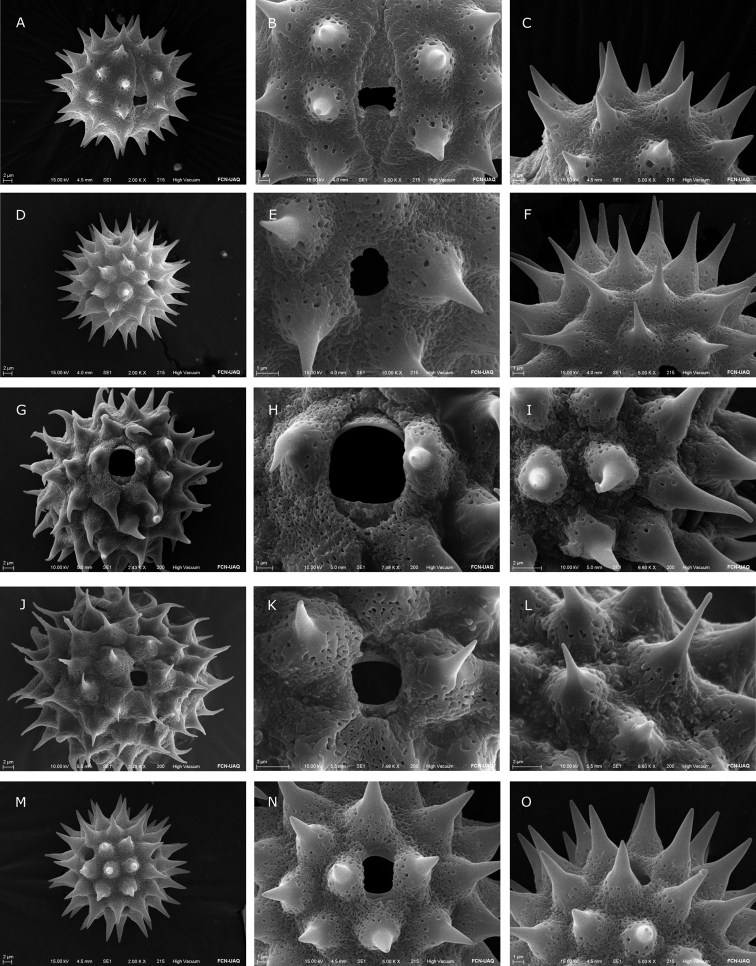
Scanning Electron Microscope (SEM) images of *Dahlia* and *Hidalgoa* pollen grains **A–C***Hidalgoaternata***A** equatorial view **B** detail of colpus **C** detail of spine **D–F***Dahliaaustralis***D** polar view **E** detail of colpus **F** detail of spine **G–I***Dahliacuspidata***G** equatorial view **H** detail of colpus **I** detail of spine **J–L***Dahlianeglecta***J** equatorial view **K** detail of colpus **L** detail of spine **M–O***Dahliacoccinea***M** equatorial view **N** detail of colpus **O** detail of spine.

**Figure 4.**
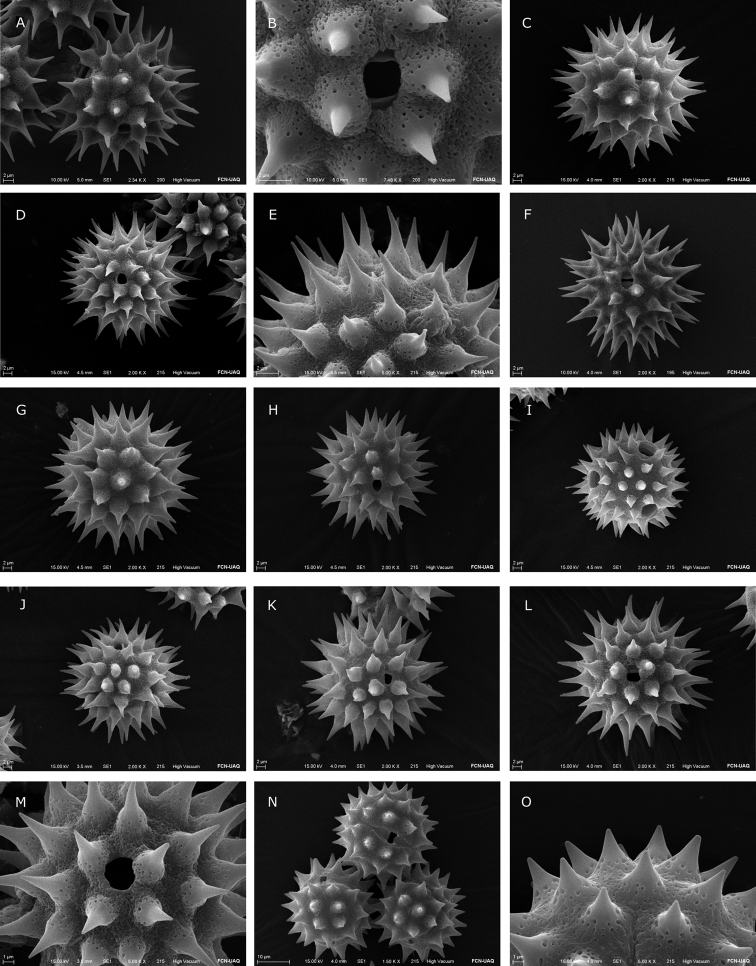
Scanning Electron Microscope (SEM) images of *Dahlia* and *Hidalgoa* pollen grains **A, B***Dahliaatropurpurea***C***Dahliabarkerae***D, E***Dahliabrevis***F***Dahliadissecta***H***Dahliaimperialis***I***Dahlialinearis***J***Dahliamerckii***K***Dahliascapigera***L***Dahliatenuicaulis***M***Dahliawixarika***N–O***Hidalgoapentamera*.

Pollen grains from a total of 25 species belonging to *Dahlia* (23 species) and *Hidalgoa* (2 species) were analyzed. Table 2 summarizes measurements and character states and Figs 2, 3 and 4 show the diversity in their pollen morphology.

The majority of species analyzed in *Dahlia* are tricoloporate. However, two out of ten pollen grains are hexacolporate in *D.cuspidata*, *D.dissecta*, *D.imperialis*, *D.merckii*, *D.parvibracteata*, *D.rudis* and *D.sorensenii*, with three apertures on one hemisphere and three on the other hemisphere (Fig. 2U). The shape is spheroidal-oblate, spheroidal or spheroidal-prolate (P/E = 0.90–1.04) and radially symmetric. Pollen size is P = 25.3 (31) 39.4 μm, E = 23.8 (31.6) 39.8 μm (Fig. 6A), and corresponds to a medium grain (Erdtman 1969). The ora are rarely lalongate, and situated distally from the equator, length 1.04 (2.51) 5.41 μm, and width 1.27 (3.60) 8.61 μm (Figs 2B, 2F, 6B), rarely acute. Colpus usually short, almost equal to ora length, more or less oval to oblong, length 2.24 (4.81) 9.2 μm, and width 1.27 (2.85) 5.89 μm (Figs 3E, 3H, 3K, 3N, 4I, 4M, 6C), apices obtuse to acute. Exine thickness thin, excluding spines, ranging from 1.1 (2.59) to 5.6 μm (Fig. 2). Ornamentation echinate; spines 4 (6–7) 10/100 μm^2^, spine length from 4.2 (7.75) to 12.26 μm, and width at base from 3.06 (6.40) to 10.35 μm, shape of spines more deltate than conical (Fig. 6D), with a distended or narrower base and with acuminate apex (Figs 3, 4). Tectum with the base of the spine always microperforate.

Pollen grains in the species of *Hidalgoa* analyzed are tricolporate and spheroidal-prolate (P/E = 1.02–1.04), radially symmetric. Pollen size is P = 25.7 (29.1) 34 μm, E = 25.1 (28.5) 32 μm (Fig. 6A), and corresponds to a medium grain (Erdtman 1969). The lalongate ora length 1.71 (2.47) to 4.87 μm, and width 6.32 (9.39) to 13.02 μm (Figs 2W, 6B), usually wider than longer and with acute apices. The colpus is elliptical, length 11.28 (14.76) to 16.83 μm, and width 2.69 (3.31) to 4.21 μm (Fig. 6C), apex always acute (Figs 3A, 3B, 4N). Exine is thinner, 1.12 (1.74) 2.95 μm excluding the spines. Ornamentation is echinate; spines 4–5/100 μm^2^, spine length ranging from 4 (5.6) to 6.77 μm and width at base 3.6 (5.3) to 7.3 μm, shape of spines conical (Figs 3C, 4O, 6D), with apex acute. Tectum with base of spine always microperforate. Palynological characters of the two studied species of *Hidalgoa* are similar, only a slight variation in pollen grain size was detected.

**Figure 5.**
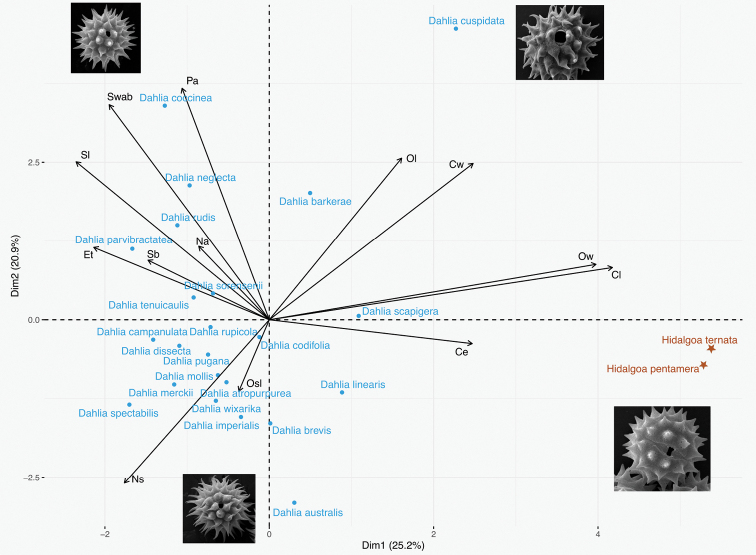
Principal Components Analysis graph showing contribution of the thirteen attributes to explain variation in pollen grains of the studied *Dahlia* and *Hidalgoa* species. **Pa** Polar axis, **Et** Exine thickness, **Cl** Colpus length, **Cw** Colpus width, **Ol** Os length, **Ow** Os width, **Sl** Spine length, **Swab** Spine width at base, **Na** Number of apertures, **Ns** Number of spines/100 μm^2^, **Ce** Colpus ends, **Sb** Base of spine, **Osl** Os lalongate.

Results of the PCA indicate that the first two components explain 46.04% of the observed variation (see Table 3 to for the contribution of each variable to Dim 1 and Dim 2). A bidimensional projection of the axes of the two first components is displayed in Fig. 5. The first principal component explains 25.19% of the variation and is associated with colpus length (Cl) and os width (Ow). The second principal component explains 20.85% and variables that contributed the most are polar axis (Pa), spine width at base (Swab), spine length (Sl), colpus width (Cw), os length (Ol), number of spines/100 μm^2^ (Ns). Length of arrows in Fig. 5 suggests adequate sampling for all characters, except for lalongate os (Osl), number of apertures (Ap) and base of spine (Sb).

## ﻿Discussion

The pollen grains of the 25 species of *Dahlia* and *Hidalgoa* we studied share the pollen type common to tribe Coreopsideae: more or less spheroidal, round in both views, tricolporate, ora lalongate, tectum microperforate, echinate, spines irregularly distributed, conical to long-pointed and smooth or distended bases with perforations (Blackmore et al 2009). The pollen of the species studied is quite homogeneous, with little variation in size and shape. Pollen grains size ranges from 25.3 to 39.4 μm in *Dahlia* and 25.7 to 34 μm in *Hidalgoa*, and the ratio of polar axis and equatorial diameter is 0.90–1.04 (*Dahlia*: 0.90–1.04, *Hidalgoa*: 1.02–1.04). The largest grains were observed in *D.neglecta* and the smallest in *D.australis* and *H.pentamera*. Based on the classification proposed by Erdtman (1969), the pollen of both genera corresponds to medium-sized grains (25–50 μm), like those described by Tellería (2017) for tribe Coreopsideae. Pollen grains in *Dahlia* and *Hidalgoa* are radially symmetrical, isopolar, and mostly spheroidal, similar to those described in *Coreopsis* (Tadesse et al. 1995).

**Figure 6.**
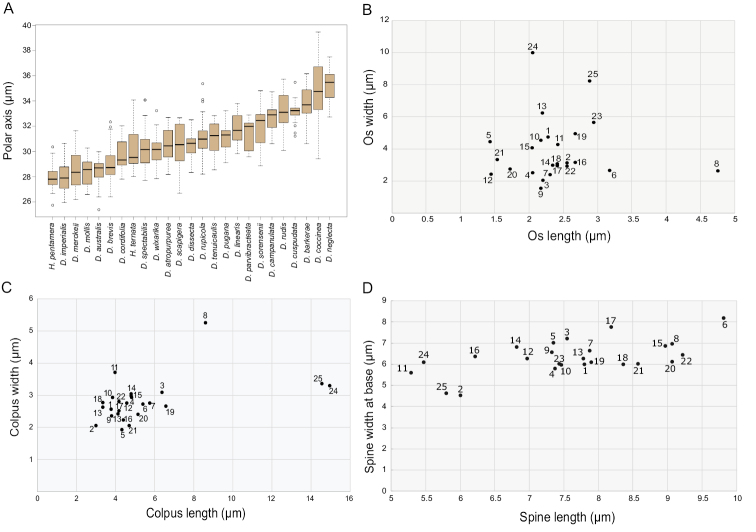
Comparison of *Dahlia* and *Hidalgoa* pollen grains **A** box plot of Polar diameter **B** os length/width **C** colpus length/width **D** spine length/width at base. **1***Dahliaatropurpurea*. **2***Dahliaaustralis*. **3***Dahliabarkerae*. **4***Dahliabrevis*. **5***Dahliacampanulata*. **6***Dahliacoccinea*. **7***Dahliacordifolia*. **8***Dahliacuspidata*. **9***Dahliadissecta*. **10***Dahliaimperialis*. **11***Dahlialinearis*. **12***Dahliamerckii*. **13***Dahliamollis*. **14***Dahlianeglecta*. **15***Dahliaparvibracteata*. **16***Dahliapugana*. **17***Dahliarudis*. **18***Dahliarupicola*. **19***Dahliascapigera*. **20***Dahliasorensenii*. **21***Dahliaspectabilis*. **22***Dahliatenuicaulis*. **23***Dahliawixarika*. **24***Hidalgoapentamera*. **25***Hidalgoaternata*.

Apertures are the most variable attribute between *Dahlia* and *Hidalgoa*, but not within the genera. Pollen in *Hidalgoa* is always tricolporate, while in pollen grains of *Dahlia*, the aperture varies from tricolporate to hexacolporate. Hexacolporate species observed here were: *D.cuspidata*, *D.dissecta*, *D.imperialis*, *D.merckii*, *D.parvibracteata*, *D.rudis* and *D.sorensenii*. Wodehouse (1930) recognized the same pattern in *D.brevis*, *D.coccinea*, *D.pinnata* and *D.imperialis*. However, Wodehouse (1930) described these six apertures as uniform in all pollen grains; the observations were made on species belonging to the San Francisco *Dahlia* Society, plants that are probably of hybrid origin. The specimens collected from the herbariums for this study do not display characters of hybrid origin. Furthermore, hexacolporate grains have been indeed reported in Old World Vernonieae and *Adenanthemum* (Blackmore et al 2009). Other members of Coreopsideae such as *Bidens* also vary in the number of colpi from 3–4 (Tadesse et al. 1995) or polypentoporate (Younis et al. 2020).

The os and colpus displayed more variation in *Dahlia* than in *Hidalgoa*. In *Hidalgoa* the os is lalongate, the widest is up to 13 μm width, and the longest colpus is approximately 17 μm, with apices always acute. These traits of *Hidalgoa* are similar to those observed in *Bidens* (Tadesse et al. 1995). In *Dahlia* the ora are either lalongate or lolongate. The os is slightly wider than larger and apices are obtuse, rarely acute. The widest and largest colpus was observed in *D.cuspidata* (5.89 μm and 9.2 μm respectively) and *D.scapigera* (ca. 7 μm), and the smallest in *D.australis* (2.9 μm). Variation in the apertures like those of *Dahlia* has not been reported in other genera of the tribe Coreopsideae (Blackmore et al. 2009).

Spines are variable between *Dahlia* and *Hidalgoa*. In *Hidalgoa* they are conical and smaller (4.08 to 6.77 μm) while in *Dahlia* they are deltate and larger (4.29 to 12.26 μm), with exception of *D.linearis* (smaller). Spines in *Dahlia* varied more in shape and size. They are commonly triangular or deltate with a broadened base (distended base), as described by Tellería (2017) for tribe Coreopsideae. Sometimes spines emerge abruptly from the exine surface, e.g. *D.campanulata*, *D.imperialis*, *D.parvibracteata*, *D.scapigera*. These spines are similar to those described in *Coreopsis* (Tadesse et al. 1995). The transition between the microperforate basal portion of the spine and the unperforated apical portion is abrupt in almost all species, except in *D.cordifolia*, *D.linearis* and *D.sorensenii*. Exine thickness did not vary among *Hidalgoa* and *Dahlia* species. The thinnest was observed in *D.cuspidata* and *D.sorensenii* (1.6 μm) and the thickest in *D.neglecta* (4.2 μm).

Multivariate analyses did not reveal a clear clustering among species of *Dahlia* according to the sections proposed by Sørensen (1969), based mainly on life form and in the phylogeny of Saar et al. (2003). Nevertheless, *D.cuspidata* and the *Hidalgoa* species are significantly different from the other *Dahlia* species included in this study. *Hidalgoa* species have long colpi and wide ora, and *D.cuspidata* has long ora. *Dahliacuspidata* possesses unusual morphological characters such as large involucral outer bracts and cuspidate leaf shape. Thus, further research might decide the position of this species.

The most recent phylogeny that included *Dahlia* and *Hidalgoa* (Sánchez-Chávez et al. 2019) identified *Hidalgoa* within the *Dahlia* clade. However, both genera are morphologically complex (Sørensen 1969; Turner 2010), and contrasting characters such as life form, number and arrangement of fertile and sterile flowers have been used to separate these two groups. A further phylogeny, including all species, may help us to better understand pollen evolution in the genus.

## ﻿Conclusions

The palynological descriptions for *Hidalgoa* presented here are the first, and despite the similarities in its floral morphology to that of *Dahlia*, its pollen is remarkably different, mostly in colpus length and shape of their spines. *Hidalgoa* has pollen grains with large colpi and small, conical spines. In addition, the length of the lalongate ora differ. Hexacolporate grains with a distended base, were found in a number of *Dahlia* species but have not been identified in *Hidalgoa*. Likewise, morphological characters such as pistillate fertile ray florets, cypselae with two apical lateral cusps and twisting petioles in *Hidalgoa* contrast with the ray florets, which can be fertile, pistillate or sterile, cypselae with pappus absent or present with 2(5) small teeth or of two weak filiform, caducous bristles of *Dahlia*; characters that have been utilized to tell these two genera apart. The results obtained in this palynological study support the idea that pollen morphology is useful for delimitation at the generic level in the *Dahlia* clade. These differentiating attributes in pollen morphology in the species of *Dahlia* and *Hidalgoa* indicate that they should be recognized as separate genera. However, as indicated above, additional anatomical and molecular characters are needed to make the taxonomic decision and help us understand evolution in the genera, and their relationship to other genera in Coreopsideae.

## Supplementary materials

Supplementary material 1 Tables S1, S2 Data type Tables (docx. file) Explanation note **Table S1**. *Dahlia* diversity. Sections proposed by Sorensen (1969). **Table S2**. Data matrix showing the values found for the 13 characters (columns) in the 25 species (rows) of *Hidalgoa* and *Dahlia* included in this study. (Characters designated according to Table 3). https://binary.pensoft.net/file/695789This dataset is made available under the Open Database License (http://opendatacommons.org/licenses/odbl/1.0/). The Open Database License (ODbL) is a license agreement intended to allow users to freely share, modify, and use this Dataset while maintaining this same freedom for others, provided that the original source and author(s) are credited.Erandi Sánchez-Chávez, Andrew Vovides, Victoria Sosa
