# Supplementary material for: ﻿Pollen morphology of the genera Hidalgoa and Dahlia (Coreopsideae, Asteraceae): implications for taxonomy
Source: PhytoKeys. 2022 Jun 13;199:187–202. doi: 10.3897/phytokeys.199.79501 (PMC9849023; doi:10.3897/phytokeys.199.79501)
Supplement: Supplementary material 1 — Tables S1, S2 [file phytokeys-199-187_article-79501__-s001.docx]

**Table S1.** *Dahlia* diversity. Sections proposed by Sorensen (1969)

| Secction | Specie |
| --- | --- |
| Dahlia | *Dahlia apiculata* |
|  | *Dahlia atropurpurea* |
|  | *Dahlia australis* |
|  | *Dahlia barkerae* |
|  | *Dahlia brevis* |
|  | *Dahlia calzadana* |
|  | *Dahlia coccinea* |
|  | *Dahlia cordifolia* |
|  | *Dahlia cuspidata* |
|  | *Dahlia hintonii* |
|  | *Dahlia hjertingii* |
|  | *Dahlia merckii* |
|  | *Dahlia mollis* |
|  | *Dahlia moorei* |
|  | *Dahlia neglecta* |
|  | *Dahlia parvibracteata* |
|  | *Dahlia pteropoda* |
|  | *Dahlia pugana* |
|  | *Dahlia purpusii* |
|  | *Dahlia rudis* |
|  | *Dahlia scapigera* |
|  | *Dahlia sherffii* |
|  | *Dahlia sorensenii* |
|  | *Dahlia spectabilis* |
|  | *Dahlia tamaulipana* |
|  | *Dahlia tenuis* |
|  | *Dahlia tubulata* |
|  | *Dahlia wixarika* |
| Entemophyllon | *Dahlia congestifolia* |
|  | *Dahlia dissecta* |
|  | *Dahlia foeniculifolia* |
|  | *Dahlia linearis* |
|  | *Dahlia mixtecana* |
|  | *Dahlia rupicola* |
|  | *Dahlia scapigeroides* |
|  | *Dahlia sublignosa* |
|  | *Dahlia mixtecana* |
| Epiphytum | *Dahlia macdougallii* |
| Pseudodendron | *Dahlia campanulata* |
|  | *Dahlia imperialis* |
|  | *Dahlia tenuicaulis* |

**Table S2.** Data matrix showing the values found for the 13 characters (columns) in the 25 species (rows) of *Hidalgoa* and *Dahlia* included in this study. (Characters designated according to Table 3).

| Specie | Pa | Et | Cl | Cw | Pw | Pl | Sl | Swab | Na | Ns | Ce | Sb | Ol |
| --- | --- | --- | --- | --- | --- | --- | --- | --- | --- | --- | --- | --- | --- |
| *Dahlia atropurpurea* | 30.43 | 2.88 | 3.79 | 2.55 | 3.12 | 2.28 | 7.80 | 5.99 | 0 | 7 | 1 | 1 | 2 |
| *Dahlia australis* | 28.43 | 2.63 | 3.01 | 2.05 | 3.08 | 2.56 | 6.01 | 4.51 | 0 | 7 | 1 | 1 | 1 |
| *Dahlia barkerae* | 33.78 | 3.58 | 6.38 | 3.09 | 5.63 | 2.20 | 7.55 | 7.21 | 0 | 4 | 1 | 1 | 1 |
| *Dahlia brevis* | 29.04 | 1.81 | 4.83 | 2.93 | 4.74 | 2.06 | 7.37 | 5.79 | 0 | 9 | 0 | 1 | 1 |
| *Dahlia campanulata* | 32.84 | 2.24 | 4.34 | 1.92 | 1.54 | 1.44 | 7.35 | 6.99 | 0 | 6 | 0 | 0 | 0 |
| *Dahlia coccinea* | 34.74 | 3.05 | 5.42 | 2.71 | 4.53 | 3.18 | 9.81 | 8.17 | 0 | 5 | 0 | 1 | 2 |
| *Dahlia codifolia* | 29.64 | 2.96 | 5.75 | 2.74 | 4.26 | 2.32 | 7.87 | 6.64 | 0 | 6 | 0 | 0 | 1 |
| *Dahlia cuspidata* | 33.16 | 1.64 | 8.61 | 5.24 | 6.22 | 4.75 | 9.07 | 6.94 | 1 | 5 | 0 | 1 | 0 |
| *Dahlia dissecta* | 30.55 | 2.88 | 3.80 | 2.35 | 2.95 | 2.18 | 7.33 | 6.57 | 1 | 6 | 0 | 0 | 2 |
| *Dahlia imperialis* | 28.04 | 1.98 | 3.85 | 2.93 | 2.93 | 2.17 | 7.46 | 5.95 | 1 | 7 | 0 | 0 | 2 |
| *Dahlia linearis* | 31.82 | 2.19 | 3.98 | 3.70 | 4.44 | 2.42 | 5.29 | 5.59 | 0 | 9 | 0 | 0 | 0 |
| *Dahlia merckii* | 28.54 | 3.05 | 4.59 | 2.75 | 3.16 | 1.45 | 6.97 | 6.25 | 1 | 7 | 0 | 1 | 0 |
| *Dahlia mollis* | 28.38 | 1.88 | 4.14 | 2.42 | 2.42 | 2.20 | 7.79 | 6.27 | 0 | 6 | 0 | 1 | 0 |
| *Dahlia neglecta* | 35.06 | 4.20 | 4.81 | 3.03 | 3.34 | 2.35 | 6.82 | 6.80 | 0 | 4 | 0 | 1 | 0 |
| *Dahlia parvibracteata* | 31.29 | 3.11 | 4.82 | 2.97 | 2.97 | 2.05 | 8.97 | 6.86 | 1 | 7 | 0 | 1 | 0 |
| *Dahlia pugana* | 31.09 | 3.42 | 4.41 | 2.23 | 4.08 | 2.68 | 6.22 | 6.35 | 0 | 7 | 0 | 1 | 2 |
| *Dahlia rudis* | 33.29 | 3.06 | 4.19 | 2.50 | 2.50 | 2.41 | 8.19 | 7.74 | 1 | 7 | 1 | 0 | 0 |
| *Dahlia rupicola* | 31.16 | 1.71 | 3.36 | 2.76 | 2.76 | 2.41 | 8.36 | 5.99 | 0 | 6 | 0 | 1 | 0 |
| *Dahlia scapigera* | 30.56 | 2.15 | 6.58 | 2.65 | 2.65 | 2.68 | 7.90 | 6.09 | 0 | 5 | 1 | 0 | 0 |
| *Dahlia sorensenii* | 31.90 | 1.68 | 5.17 | 2.40 | 2.40 | 1.73 | 9.07 | 6.12 | 1 | 5 | 0 | 0 | 0 |
| *Dahlia spectabilis* | 30.33 | 2.46 | 4.72 | 2.05 | 2.05 | 1.54 | 8.57 | 6.00 | 0 | 7 | 0 | 1 | 2 |
| *Dahlia tenuicaulis* | 31.03 | 2.82 | 4.19 | 2.79 | 4.94 | 2.56 | 9.22 | 6.43 | 0 | 7 | 0 | 1 | 2 |
| *Dahlia wixarika* | 30.12 | 2.42 | 3.36 | 2.63 | 2.63 | 2.95 | 7.44 | 6.01 | 0 | 8 | 0 | 0 | 2 |
| *Hidalgoa pentamera* | 27.97 | 1.78 | 14.97 | 3.29 | 9.98 | 2.06 | 5.47 | 6.09 | 0 | 5 | 1 | 0 | 1 |
| *Hidalgoa ternata* | 30.36 | 1.71 | 14.55 | 3.34 | 8.21 | 2.89 | 5.80 | 4.63 | 0 | 4 | 1 | 0 | 1 |
